# Effect of 4 weeks of plyometric training in the pre-competitive period on volleyball athletes’ performance

**DOI:** 10.5114/biolsport.2023.112971

**Published:** 2022-02-18

**Authors:** Miller P. Guimarães, Rodrigo D. O. Silva, Igor A. Dos Santos, Gaspar P. Da Silva, Yuri A. C. Campos, Sandro F. Da Silva, Paulo Henrique S. M. De Azevedo

**Affiliations:** 1 Grupo de Estudos e Pesquisas em Fisiologia do Exercício, Universidade Federal de São Paulo, Santos, Brasil; 2 Programa de Pós-graduação em Ciências do Movimento Humano e Reabilitação, Universidade Federal de São Paulo, Santos, Brasil; 3 Faculdade Presbiteriana Gammon, Lavras, Brasil; 4 Grupo de Estudos e Pesquisas em Respostas Neuromusculares, Universidade Federal de Lavras, Lavras, Brasil; 5 Programa de Pós Graduação da Faculdade de Educação Física e Esportes, Universidade Federal de Juiz de Fora, Juiz de Fora, Brasil; 6 Centro Mineiro do Ensino Superior, Campo Belo, Brasil

**Keywords:** Sports, Muscle power, Countermovement jump, Training, Season

## Abstract

We aimed to evaluate the effect of 4 weeks of plyometric training (PT), performed in the pre-competitive period, on the vertical jump performance of professional volleyball athletes. We recruited 17 professional female volleyball players (age: 19 ± 3 years; weight: 67.2 ± 5.50 kg; height: 1.81 ± 0.22 m; body fat: 14.4 ± 2.12%; squat 1RM test: 75.5 ± 7.82 kg; training time experience: 6.2 ± 3.4 years) to participate in four weeks of training and assessments. They were divided into an experimental group (EG = 9) and a control group (CG = 8). Both groups were submitted to friendly matches, technical, tactical and resistance training (4 weeks/˜9 sessions per week), and internal load monitoring was carried out. The EG performed PT twice a week. At the beginning and end of the four weeks, jump tests were performed. The main findings are: 1) PT when incorporated into the pre-competitive period can induce greater improvements in jumping performance (EG = 28.93 ± 3.24 cm to 31.67 ± 3.39 cm; CG = 27.91 ± 4.64 cm to 28.97 ± 4.58 cm; when comparing the percentage delta, we found a difference between groups with ES of 1.04 and P = 0.02); 2) this result is observed when the training load is similar between groups and increases over the weeks, respecting the linear progression principle. Therefore, including plyometric training in the preparatory period for volleyball, with low monotony and training strain increment, is an effective strategy for further CMJ performance improvement.

## INTRODUCTION

Different training methods are applied across the training periods in a periodization [1]. In the pre-competitive period, there is a predominance of high training volume. As the training progresses, the volume is decreased and the intensity is increased [1]. Therefore, power training, such as plyometrics, is generally included in the competitive training period [2]. This logical and linear sequence, represented by the decrease in volume and increase in intensity, is applied to reduce the chance of concurrent training [1, 3]. However, the short time of the pre-competitive period and the most frequent game schedule [3] mean that volleyball athletes need to improve their jumping performance from the pre-competitive period on [4].

The motor pattern of countermovement jump (CMJ) movements is essential to serve, block and attack in volleyball [5], as well as for performance assessment [6, 7]. Thus, using jumps at specific periods in a season (such as the pre-competitive period) can help physical trainers and coaches to quantify the training programme efficacy performance improvement [8]. To improve jumping performance, plyometric training (PT) (exercises that are characterized by a rapid transition between the eccentric and concentric phases, generating greater accumulation of elastic energy) [2, 9] is a valid method [4, 10, 11]. However, there are still gaps in the literature regarding the efficacy of PT applied in the pre-competitive period and whether it promotes the concurrent effect.

The internal and external training load monitoring must be controlled for performance improvement [5, 12–14]. Training monitoring through rating of perceived exertion (RPE) and session duration (in minutes) is an effective and inexpensive way to quantify the training impulse [15]. The session’s RPE, total weekly training load (TWTL), monotony and training strain are examples of these variables [5, 12, 14]. Here we evaluate whether the inclusion of plyometric training during the pre-competitive period has a concurrent effect with technical and tactical training, friendly matches and resistance training or if it contributes to the jump performance improvement.

Therefore, the aim of the present study was to evaluate the effect of 4 weeks of PT, performed in the pre-competitive period, on the jump performance of the professional female volleyball athletes.

## MATERIALS AND METHODS

### Study design

The sample consisted of 17 female players ([mean ± SD] age: 19.5 ± 3.73 years; weight: 67.2 ± 5.50 kg; height: 1.81 ± 0.22 m; body fat: 14.2 ± 2.53%; 1RM test: 75.5 ± 7.82 kg; training time: 6.2 ± 3.4 years), randomly divided into an experimental group (EG): n = 9 and a control (CG): n = 8 (with practically the same number of athletes per position within each group), belonging to a Brazilian professional club, participating in Superliga B, in the year 2018. The evaluations were carried out in 4 days of nonconsecutive tests. The first visit to the laboratory was for familiarization with the maximum repetition test (1RM), CMJ, RPE scale and training protocols, randomization and anthropometric data collection. After 48 hours, the second visit was for the 1RM test. Again, after 48 hours, the third visit was for the pre-intervention CMJ test. Between the third and fourth visits to the laboratory, the EG and CG four-week training programmes were carried out, with the internal load being monitored by the RPE of the session and total training time (expressed in TWTL), monotony and strain. Finally, after four weeks of intervention, the fourth visit was for the post-intervention CMJ test and anthropometric data. The CMJ tests were performed on Monday morning of the first week of intervention and after the four weeks of intervention. With this procedure, we ensure that athletes come from a more extended period of rest to the detriment of the weekend. All athletes were already familiarized with the instruments used in the research.

The volunteers were informed about the purpose of the study and signed the Informed Consent Form following the recommendations of Resolution 466/12 of the National Health Council. The Research Ethics Committee approved the study.

### Training protocol

The study was carried out within the pre-competitive period and lasted for four weeks. The mesocycle schedule consisted of technical/ tactical training sessions, friendly games and resistance training (RT), in addition to PT for EG. The distribution of training sessions over the four weeks can be seen in Table 1. The technical/tactical training models were based on the study by Trajkovic [16]. The RT consisted of performing exercises that were already part of the athletes’ training routine (an organization aimed at recruiting the whole body, in exercises such as squat with body weight, prone bridge and hip thrust with body weight, adduction and abduction with elastic bands, open row with elastic bands and push-up exercise). Four sets (60 seconds rest) were performed for each exercise with open repetitions/durations. The orientation was for the intensity of each series not to exceed 7 in the RPE and 3 in the total session. The objective of these sessions was to maintain the general conditioning already proposed in the two previous weeks. For both groups, given the specificities of each position in tactical and technical training, similar total training loads (external load) were applied and the internal load was recorded. PT sessions for the EG were held while the CG performed technical and tactical training. Before all training sessions, the two groups completed a warm-up for approximately ten minutes consisting of light running, dynamic stretches, and general movements.

**TABLE 1:** Training sessions distributions in the intervention period.

Day	Period	Week 1–2	Week 3	Week 4
Monday	Morning	CMJ + TTT	TTT	TTT
	Afternoon	TTT	TTT	TTT
Tuesday	Morning	PT(EG)/TTT(CG)	PT(EG)/TTT(CG)	PT(EG)/TTT(CG)
	Afternoon	OFF	OFF	TTT
Wednesday	Morning	RT + TTT	TTT	TTT
	Afternoon	TTT	TTT	TTT
Thursday	Morning	PT(EG)/TTT(CG)	PT(EG)/TTT(CG)	PT(EG)/TTT(CG)
	Afternoon	OFF	OFF	OFF
Friday	Morning	TTT	TTT	TTT
	Afternoon	OFF	TTT	TTT
Saturday	Morning	TTT	OFF	OFF
	Afternoon	OFF	FM	FM
Sunday	Morning	OFF	OFF	OFF
	Afternoon	OFF	OFF	OFF

N sessions		8	9	10

Legend: PT: Plyometric Training; RT: Resistance Training; TTT: Technical/Tactical Training; FM: Friendly Matches; CMJ: countermovement jump Test; EG: Experimental Group; CG: Control Group.

Two weeks before the intervention, the players underwent technical/tactical, aerobic, strength-endurance, balance, and flexibility training, in addition to neuromuscular assessments. The aim of those two weeks was to improve the general conditioning of all players as well as to avoid injuries [1, 16]. The internal load monitoring (ex-pressed in TWTL) from this period was also recorded and classified as having moderate-low magnitudes (≥ 1257 to < 2514 arbitrary units [AU]) for both groups [12].

### Plyometric training protocol

The PT protocol consisted of 8 sets of jumps per session, with and without additional load, being: initially, four sets of 10 consecutive maximum jumps, with additional load coming from a bar positioned in the cervical region (specificity of the squat exercise), with 20% of RM. Soon after, there were 4 sets of 15 seconds each (adapted from Hespanhol, Silva Neto, and Arruda [17] of maximum consecutive jumps, without additional load and applying the CMJ pattern. The pattern of movement, for both the jumps with and without additional load, was from crouching to approximately 90º of knee flexion. For jumps with the additional load, the hands should stabilize and keep the bar resting on the cervix. Between sets and exercises, a rest interval of 90 to 120 seconds was applied. For all jumps, experienced evaluators verbally encouraged and gave feedback to the athletes about the movement pattern. In all PT sessions, all athletes were able to perform 10 jumps in the 4 sets with additional load and also maintain the CMJ movement pattern in the 4 sets of 15 seconds each. As they are professional athletes, they already had experience in plyometric training, which justifies the choice of PT.

### Research tools

### Anthropometry

To characterize the sample, we determined the height and body mass of the subjects, using a scale that had a stadiometer (110 FF, Welmy, Santa Bárbara d’Oeste, Brazil). The fat percentage was estimated using a bioimpedance device (Quantum BIA-II, RJL Systems, Clinton Township, USA). Tetrapolar electrodes were used for collection (Bio Tetronic, Sanny, São Bernardo do Campo, Brazil).

### 1RM test

The 1RM test was performed through the half squat exercise (approximately 90º of knee flexion in eccentric/concentric transition action). Initially, the participants performed a general warm-up, followed by performing a specific warm-up set that consisted of 8 repetitions at approximately 50% of the estimated 1-RM. Then they performed another series of 3 repetitions at 70% of the estimated 1-RM. After 5 minutes of recovery, load increments were applied until failure. The number of single series did not exceed 5 attempts. The rest interval between sets was 5 minutes [18]. During the test, the cadence of movement was controlled using a digital metronome (DM90, Seiko, Tokyo, Japan), with 2 seconds for the eccentric phase and 2 seconds for the concentric phase.

### Internal load monitoring

The session RPE scale, monotony and training strain were measured to control the internal load [15]. Athletes assessed psychophysiological stress through the RPE session. This value was multiplied by the duration of the training session, in minutes, and this final product represented the training load of the session (value in AU). The procedure was performed 20 to 30 minutes after each training session, for each athlete, being asked: “How was your training today?” The TWTL (sum of the loads of each training session in the weeks) the monotony (average of the training loads of the sessions weekly divided by their standard deviation) and strain (multiplication of monotony by the sum of training loads accumulated in the week) [15] were used for the analyses.

### Countermovement jump assessment (CMJ)

The participants were subjected to the CMJ test to measure the jump height performance at different times [19], using a contact platform (Cefise, Brazil) interconnected to the software (Jump System, 1.0, Brazil) [14, 20]. Before the jumps, each athlete performed a standardized 5-minute warm-up consisting of general movements and dynamic stretching. To perform the jumps, the hands were on the hips and participants were previously instructed to squat at approximately 90º of knee flexion, jump as high as possible and keep the legs straight in the flight phase. Athletes performed three vertical jumps with a 5-second rest interval between attempts. The mean value of the three attempts of CMJ was used for analysis [21]. The coefficient of variation (%) of the CMJ was 1.1 for the EG before and after intervention; 1.7 for the CG before intervention and 1.6 after intervention.

### Statistics

ANOVA for repeated measures was applied to compare TWTL, monotony and strain through the weeks. We checked the data sphericity through Mauchly’s test, and when appropriate a Greenhouse-Geisser correction was made. One-way analysis of variance (ANOVA) testing was used to compare groups at baseline for CMJ; if the F-statistic test showed a significant difference, the Bonferroni post-hoc test was applied to show the direction of the difference, but it was not necessary. The delta comparison for CMJ between groups was made through the independent t-test, and the effect size was calculated through Hedge’s g. We applied the dependent t-test to compare body mass and percentage of body fat within groups before and after 4 weeks of training. The assumption of the equality of variances was confirmed by examination of Levene’s test and normality through the Shapiro-Wilk test. The between-group difference of the change in continuous variables from baseline to 4 weeks was assessed through analysis of covariance (ANCOVA), with the week four values as the dependent variables and the baseline value as a covariate and, where appropriate, a post-hoc analysis with a comparison of main effects and a Bonferroni adjustment. Following the ANCOVA analyses the Cohen’s d test was applied to evaluate the effect size (ES) of CMJ before and after treatment. Cohen’s categories used to assess the magnitude of ES were: (small if 0 ≤ [g] ≤ 0.50; medium if 0.50 < [g] ≤ 0.80; and large if [g] > 0.80). The level of significance adopted for the analyses was p ≤ 0.05. The smallest worthwhile change (SWC) was set by using Cohen’s principles for a small ES (i.e., 0.2) for each variable tested [22]. We conducted all analysis through JASP 0.9.2.

## RESULTS

Table 2 shows the body composition of both groups at the beginning and end of the intervention, showing that there are no significant changes in any variable.

**TABLE 2:** Comparison of anthropometric data between the pre and post groups.

	Experimental Group (n = 9)				Control Group (n = 8)			
Variables	Pre Training	Post Training	*p*	E.S.	Pre Training	Post Training	*p*	E.S.
Body Mass (kg)	67.7 ± 4.32	67.5 ± 4.77	0.54	-0.04	66.9 ± 6.35	66.6 ± 5.48	0.78	-0.05
Body Fat (%)	14.4 ± 2.12	14.2 ± 1.85	0.87	-0.10	14.1 ± 2.75	14.0 ± 1.98	0.85	-0.04
Heigth (m)	1.80 ± 0.30	–	–	–	1.82 ± 0.21	–	–	–

**FIG. 1:**
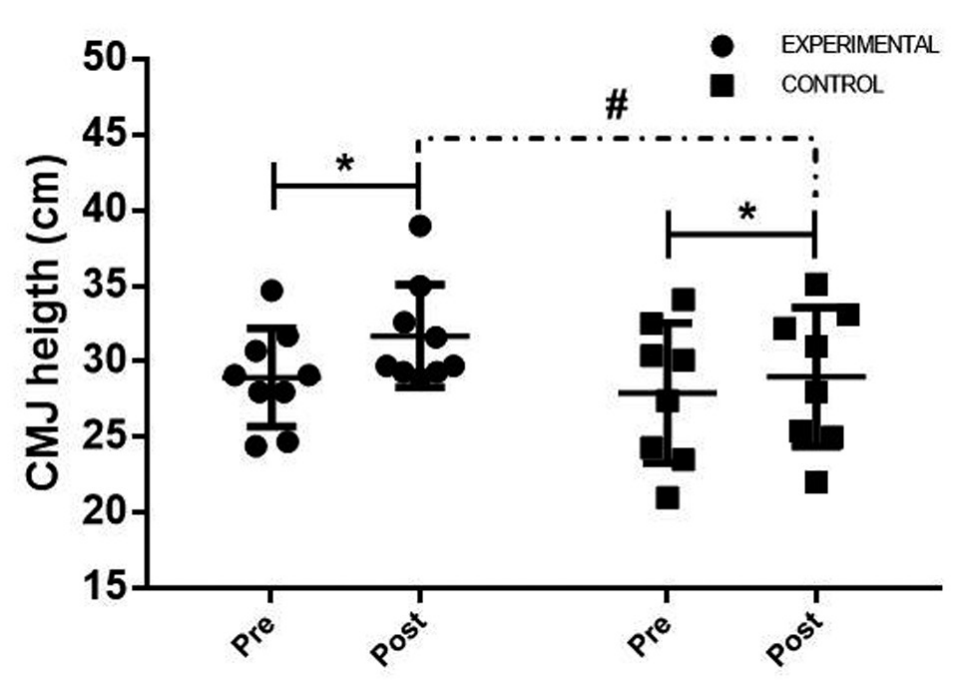
Comparison of the Countermovement Jump between and within groups * Difference between-group pre- and post-intervention moments: p≤ 0.05; # Difference within-group pre- and post-intervention moments: p≤ 0.05.

Figure 1 shows the comparisons of the within- and between-group CMJ at the pre- and post-intervention time points. The values of both groups were as follows: the EG showed an increase from 28.93 ± 3.24 cm to 31.67 ± 3.39 cm between the pre- and post-intervention measurement; the CG recorded an increase from 27.91 ± 4.64 cm to 28.97 ± 4.58 cm between the pre- and post-intervention assessment. The analysis of covariance, considering the pre-test CMJ as a covariable, found a difference between the groups for the post-training measurement (ES: 0.43; P = 0.027) with an average difference between groups of 1.74 cm. For the baseline treatment, no differences were found between groups (ES: 0.25; P = 0.60) with an average difference between groups of 1.02 cm.

**FIG. 2:**
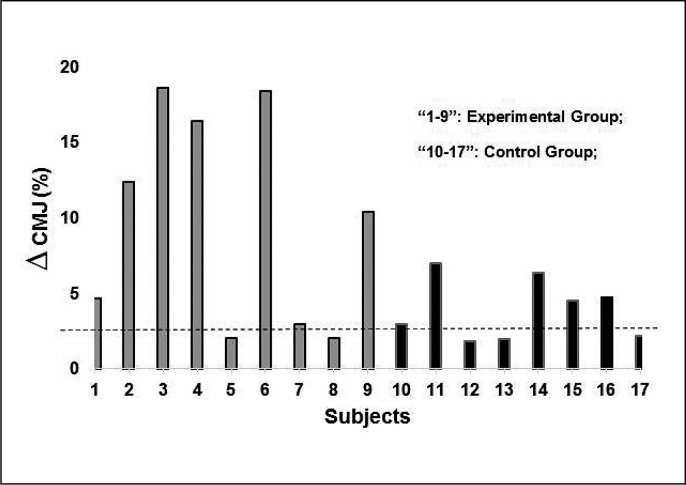
Individual plots and smallest worthwhile change of the gain values in the Countermovement Jump.

Athletes should increase the jump height by 0.77 cm (2.72%) as estimated by SWC analyses. From Figure 2, we can see that two athletes (22.2%) from the EG and three athletes (37.5%) from the CG had no significant improvement at the end of the preparatory period. The improvement of all athletes is possibly due to the internal load, monotony, and strain being within expected. However, the EG had a more considerable improvement than the CG, showing the importance of including plyometric training at this stage of training. In the graph below, grey is the EG and black is the CG. The dotted line is the SWC (2.72%).

**FIG. 3:**
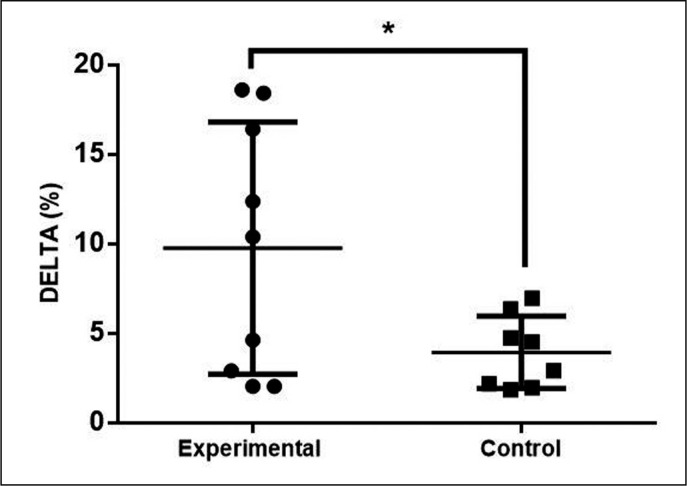
Comparison of the percentage delta Countermovement Jump between groups * Difference between-group: p≤ 0.05.

When comparing the percentage delta, we found a difference between groups (EG: 9.77 ± 2.34% and CG: 3.94 ± 0.71%) with ES of 1.04 and P = 0.02 (Figure 3).

**FIG. 4:**
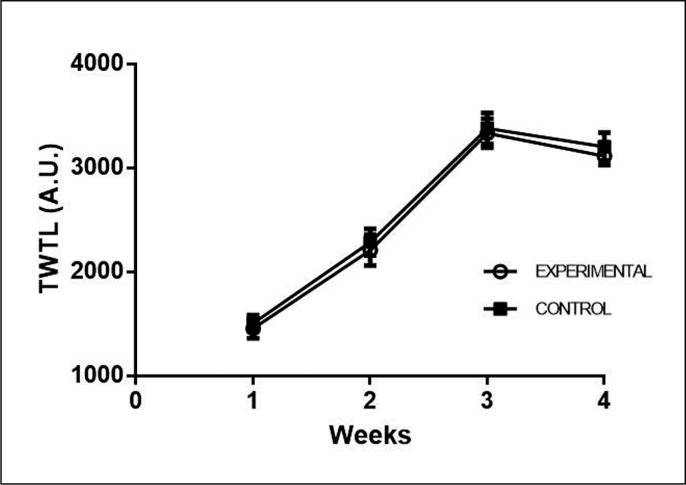
Comparison of the Total Weekly Training Load (TWTL) within and between groups during the four weeks.

Figure 4 shows the results of TWTL within and between the EG and CG during the 4 weeks of training. TWTL was not different between groups within each week. For EG, week 1 had lower TWTL than weeks 2 (ES: -3.01), 3 (ES: -7.52) and 4 (ES: -6.63) (P < 0.001). Week 2 had lower TWTL than weeks 3 (ES: -4.50) and 4 (ES: -3.62) (P < 0.001). Week 3 had higher TWTL than week 4 (ES: 0.88; P = 0.019). The CG had a similar response, with week 1 having lower TWTL than weeks 2 (ES: -2.94), 3 (ES: -7.07) and 4 (ES: -6.40) (P < 0.001). Week 2 had lower TWTL than weeks 3 (ES: -4.13) and 4 (ES: -3.46) (P < 0.001). Week 3 had higher TWTL than week 4 (ES: 0.66; P = 0.25).

**FIG. 5:**
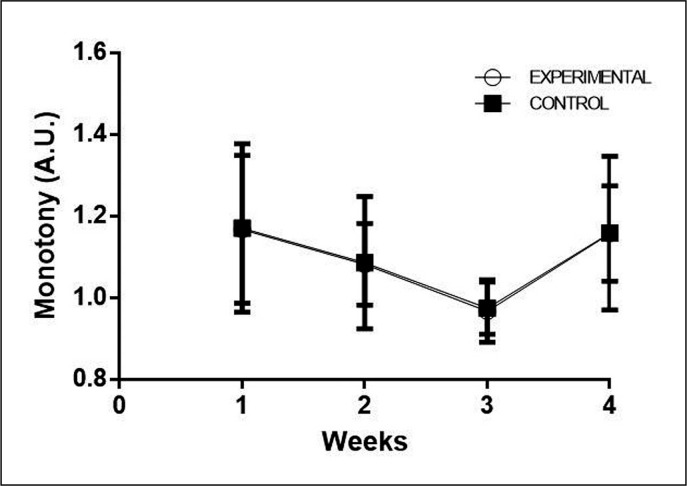
Comparison of the Monotony within and between groups during the four weeks.

As shown in Figure 5, a decrease in monotony was observed with the training progression. Week 3 of the CG had less monotony than week 1 (ES: 0.855; P = 0.028) and week 4 (ES: -0.80; P = 0.053). The same was observed for EG (ES: 0.924; P = 0.012 and ES: -0.878; P = 0.021).

**FIG. 6:**
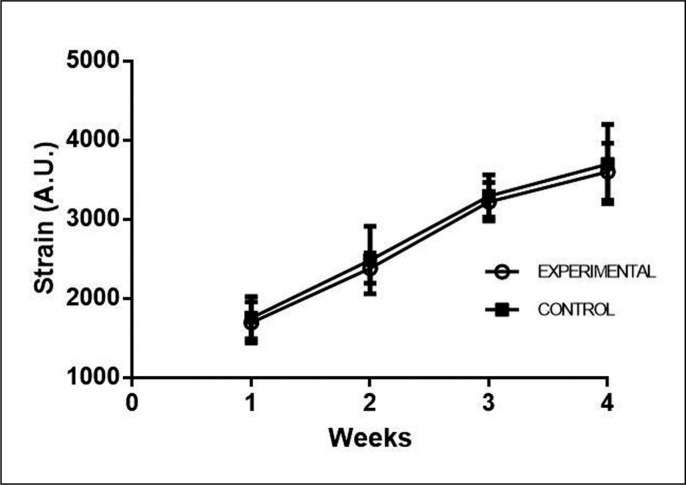
Comparison of the Strain within and between groups during the four weeks.

Finally, Figure 6 shows how the strain increased with the training progression. There was no difference between groups. Within the groups, the only time when there was no difference was between weeks 3 and 4.

## DISCUSSION

Our main findings showed that both groups improved their CMJ performance at the end of the intervention period. However, the EG achieved better results than the CG, both in values of percentage delta (P = 0.02 and ES of 1.042) and CMJ height (P = 0.027 and ES of 0.43), as well as according to the analysis of individual data and SWC (22.2% (EG) versus 37.5% (CG) of the athletes did not improve what they could have improved according to the SWC values (0.77 cm or 2.72%)). One of the initial hypotheses was that both groups could improve the CMJ performance because they were at the beginning of the season. However, there was uncertainty as regards the amount of improvement each group would achieve and whether the PT of just 4 weeks, spread over 8 sessions, would provide a more substantial effect than technical and tactical training only. Our data show that the increase in PT incorporated into the specific volleyball training in the pre-competitive period brought greater benefits to the performance of the athletes in the EG than the CG.

Another critical point to be discussed was the initial equalizations between the groups for the CMJ performance, the internal load monitoring throughout the intervention and the lack of change in body composition during the intervention as well. Such mechanisms allow us to affirm that the greatest gain obtained by the EG came from the plyometric training incorporation. It is worth mentioning that all athletes who started the study ended without any injury.

For performance variables such as the CMJ to be faithfully assessed and compared in specific periods and between groups, it is necessary to monitor the internal load. The relationship between what was proposed by the physical trainer (external load) and what was reported by athletes (internal load) was expressed in data from TWTL, monotony, and strain [5, 12, 14, 23]. Training strain data report a progression of the load over 4 weeks. Also, a monotony threshold with a value of 2.0 is proposed [24, 25], with values above that presenting a higher risk for overreaching and performance decrement. In any of the weeks the threshold value was reached for any group; a study [12] reported values of up to 1.6 for monotony, 8000 AU for strain and 4500 for TWTL in the pre-competitive period for professional volleyball players from the main Brazilian league. Thus, the data of the internal load monitoring obtained further confirm that the distribution of the external training load in the present study was suitable.

The plyometric training protocol proposed in our study suggests a load of 20% of the RM and consecutive jumps, added to an evaluation protocol proposed elsewhere [19]. Studies using the squat jump with an additional load to improve lower limb power in athletes are found in the literature [26–28]. However, they used different methodologies and adaptive physiological mechanisms from ours. Such studies propose jumping training based on the hysteresis mechanism [29], which benefits the action of the active or contractile components of the neuromuscular system. Thus, the methodology proposed here was not found in the literature, making it an efficient, applicable and unprecedented tool.

The neural and mechanical adaptations coming from the PT and achieved by the EG were in a higher magnitude than the CG. The stretching-shortening cycle is one of the primary adaptations promoted by PT and could explain athletes’ improvement in performance, through the increased muscle-tendon junction stiffness [2] and better use of elastic components [30]. Also, mechanoreceptors such as muscle spindles and Golgi tendon organs are sensitive to PT. The first one undergoes adaptations of higher stimulation [31] and the second one undergoes adaptations of inhibition [32]. This combination could provide the jump performance improvement. Consequently, intermuscular coordination and motoneuron firing frequency establish a more acute adaptive form, thus helping to improve performance. Although these described adaptations are speculative (we did not evaluate them directly, which therefore constitutes a limitation of the study), we must pay attention to some details. As they are professional athletes with great experience in performing the CMJ test (proved by the low coefficient of variation), the maintenance of the motor pattern at all times can be verified. Thus, this variable did not interfere with the results obtained. Furthermore, based on Figure 2, we can see that all athletes improved their jump height. However, the individual variation within and between groups (assessed by the SWC), even the athletes being in the same phase of the season and being randomly allocated to each group, seems to demonstrate that experienced athletes (in the pre-competitive period and lasting only 4 weeks of training) benefit in greater magnitude from the PT, which is the cause of the greatest improvement in the EG.

The short time of 4 weeks seems to offer different responses to training. Mazurek et al. [33] found no differences in CMJ height over 5 weeks for a group of handball players who performed PT twice a week. However, Debien et al. [12] verified the difference in the CMJ of professional volleyball players from 2 to 5 weeks in the pre-competition period. Additionally, in a recent study the authors applied the PT over 6 weeks on volleyball players and also observed significant improvements in the CMJ [2]. This CMJ is widely used to measure the players’ performance in athletes of different levels, having excellent applicability and reproducibility [34, 35]. The specific jump training that contains sports movements seems to be more useful for performance evaluation [36]. Therefore, the usage and performance values of the CMJ observed here are quite similar to data obtained in another study (˜30 cm) with women athletes from the first and second Slovenian and Portuguese volleyball divisions, showing that our findings are consistent with those of the current literature.

Finally, the outcomes from the applied PT in the pre-competitive period reported here increase the inquiries regarding the classic linear periodization. Thus, having established the effectiveness of a PT programme at different periods of the season [2, 4], it is clear that the results obtained from the CMJ performance and also the absence of injury among the assessed players substantially increase the practical applicability of the research. It is clear that the sample size (relatively small), the length of stay in the offseason period (uncontrolled) and the extrapolation of the effects of PT by four weeks for the entire season are limitations of the study. However, coaches and fitness trainers can apply this complementary methodology especially when the season calendar suggests short pre-competition times.

## CONCLUSIONS

We conclude that plyometric training, when incorporated into specific volleyball training in the preparatory period, can induce more substantial improvements in jumping performance (˜ 10%). This result was observed when the training load was similar between groups and increased over the weeks, respecting the linear progression principle. Also, we observed a decrease in training monotony over the weeks, and strain increase, which shows that the training was well distributed. Therefore, including plyometric training in the preparatory period for volleyball, with low monotony and training strain increment, is an effective strategy for further CMJ performance improvement.
